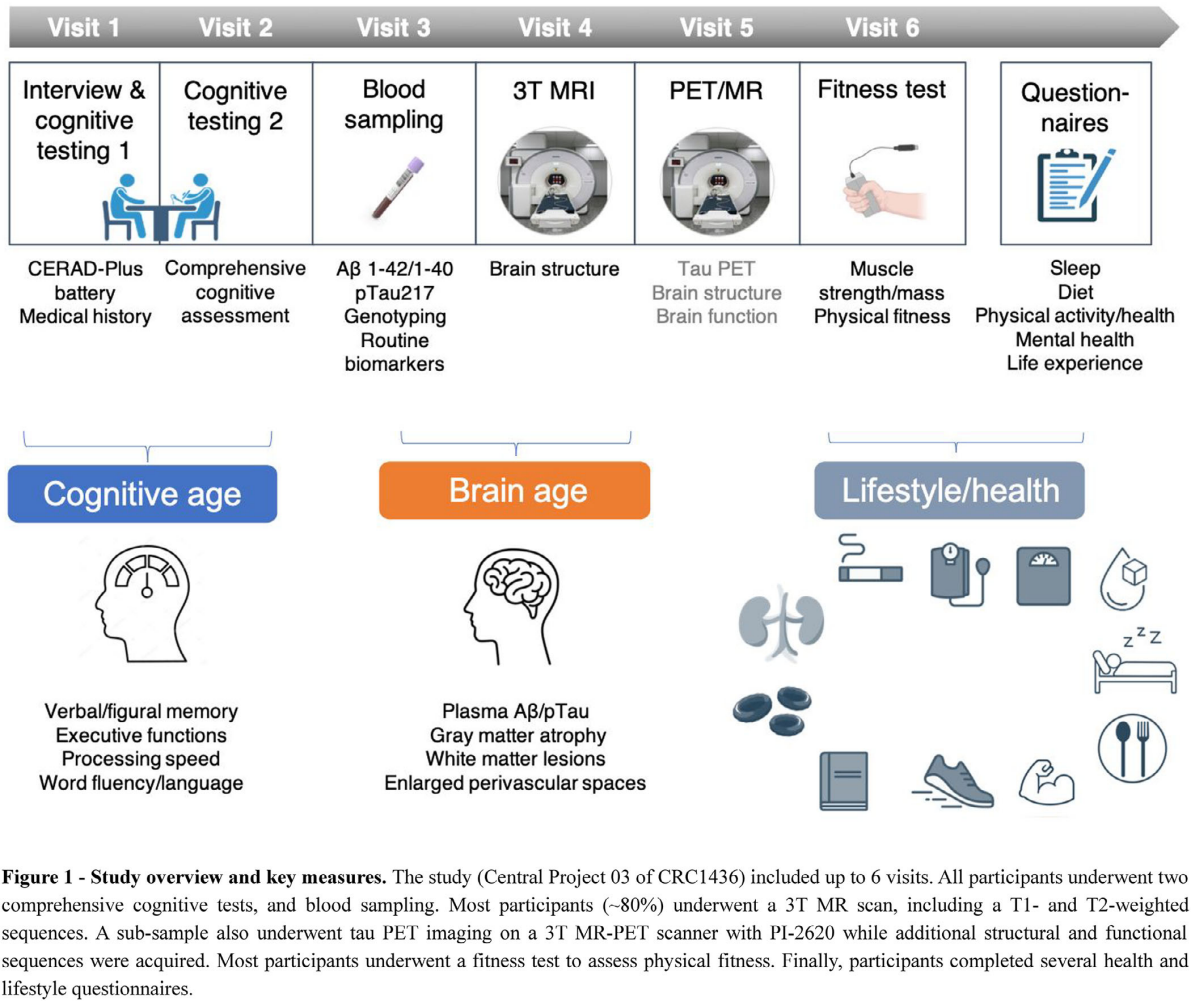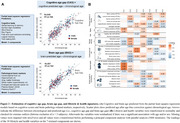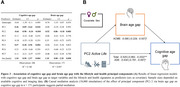# Lifestyle and health signatures of brain pathological and cognitive aging

**DOI:** 10.1002/alz70862_109741

**Published:** 2025-12-23

**Authors:** Niklas Behrenbruch, Svenja Schwarck, Beate Schumann‐Werner, Eóin N. Molloy, Anne Hochkeppler, Anna‐Therese Büchel, Jose Bernal Moyano, Enise I Incesoy, Berta Garcia‐Garcia, Niklas Vockert, Barbara Morgado, Larissa Fischer, Patrick Müller, Gusalija Behnisch, Constanze I. Seidenbecher, Björn H. Schott, Hermann Esselmann, Jens Wiltfang, Henryk Barthel, Osama Sabri, Michael C. Kreissl, Emrah Düzel, Anne Maass

**Affiliations:** ^1^ German Center for Neurodegenerative Diseases (DZNE), Magdeburg Germany; ^2^ Institute of Cognitive Neurology and Dementia Research (IKND), Otto‐von‐Guericke University, Magdeburg Germany; ^3^ Faculty of Natural Sciences, Otto‐von‐Guericke University Magdeburg, Magdeburg Germany; ^4^ Division of Nuclear Medicine, Department of Radiology & Nuclear Medicine, Faculty of Medicine, Otto von Guericke University, Magdeburg Germany; ^5^ University Clinic for Psychosomatic Medicine and Psychotherapy, Otto‐von‐Guericke University Magdeburg, Magdeburg Germany; ^6^ Centre for Clinical Brain Sciences, The University of Edinburgh, Edinburgh, Scotland UK; ^7^ Department of Psychiatry and Psychotherapy, University Medical Center Göttingen (UMG), Göttingen Germany; ^8^ German Center for Neurodegenerative Diseases (DZNE), Magdeburg, Sachsen‐Anhalt Germany; ^9^ University Hospital Magdeburg; Devision of Cardiology and Angiology, Magdeburg, Sachsen‐Anhalt Germany; ^10^ Leibniz Institute for Neurobiology (LIN), Magdeburg Germany; ^11^ German Center for Neurodegenerative Diseases (DZNE), Göttingen Germany; ^12^ Department of Psychiatry and Psychotherapy, University Medical Center Goettingen (UMG), Göttingen Germany; ^13^ Department of Nuclear Medicine, University of Leipzig, Leipzig Germany; ^14^ Institute of Cognitive Neurology and Dementia Research (IKND), Otto‐von‐Guericke University, Magdeburg, Sachsen Anhalt Germany

## Abstract

**Background:**

While aging almost inevitably leads to some degree of cognitive decline, the interindividual heterogeneity in the trajectories of decline raises the question of the extent to which resistance against pathology and cognitive resilience are involved. Using a multimodal approach including neuroimaging, fitness assessment, questionnaire data, and Alzheimer’s disease (AD) genetic risk and plasma biomarkers (Figure 1), we aimed to characterize latent structures of lifestyle, mental and bodily health, estimate indices of brain (pathological) and cognitive aging, and relate lifestyle/health profiles and AD genetic risk to these indices.

**Method:**

We analyzed a subsample of 211 cognitively normal older adults aged ≥ 60 years from an ongoing study (CRC1436) (age=71.0±7.4years, 46% female). Using principal component analysis, we derived seven principal components (PCs) that capture latent structures of lifestyle and general health from thirty variables (Figure 2B). To characterize successful brain/cognitive aging, we calculated a brain (BAG) and cognitive age gap (CAG) as the difference between brain pathology‐/cognition‐predicted age and chronological age (Figure 2A). Our novel BAG estimate incorporated also AD pathology, white matter hyperintensities and enlarged perivascular spaces. We regressed the first seven principal components (PC) on BAG and CAG to estimate the association of lifestyle/health profiles with successful brain/cognitive aging. We further assessed whether APOE4 carriers had higher BAG/CAG using a two‐sample t‐test.

**Result:**

We named the PCs according to their main factor loadings (Figure 2B). PC1 (*Low Mental Health)*, PC2 (*Active Life)*, and PC5 (*Mentally Inactive & Physically Active)* were significantly associated with CAG, whereas only PC2 was significantly associated with BAG (Figure 3A). BAG partly explained the relationship between PC2 and CAG (partial mediation of 18.0% of total effect, *p* = 0.027; Figure 3B). Finally, APOE e4 carrier had significantly higher BAG (*p* = 0.049), but not CAG (*p* = 0.155).

**Conclusion:**

Our results suggest that factors of cognitive resilience and brain maintenance are to some extent unified in an active lifestyle described by physical fitness, mental leisure activities, and lower cardiovascular risk. In addition, engagement in mental leisure activities may explain cognitive resilience independent of brain pathology. Finally, genetic risk for AD may also accelerate brain aging in cognitively healthy older adults.